# Continuous Activation of Dopamine Receptors Alleviates LPS-Induced Liver Injury in Mice via β-arrestin2 Dependent Akt/NF-κB Pathway

**DOI:** 10.3389/fphar.2022.853834

**Published:** 2022-03-14

**Authors:** Mingan Li, Ce Zhang, Lin Zhou, Xiaohui Sun, Tian Wang, Fenghua Fu

**Affiliations:** School of Pharmacy, Key Laboratory of Molecular Pharmacology and Drug Evaluation, Ministry of Education, Collaborative Innovation Center of Advanced Drug Delivery System and Biotech Drugs in Universities of Shandong, Yantai University, Yantai, China

**Keywords:** rotigotine, extended-release microsphere, dopamine receptors agonist, inflammation, lipopolysaccharide, liver injury

## Abstract

Many studies showed that dopamine receptors (DRs) agonists have anti-inflammatory effects. Rotigotine, a non-ergot dopamine receptor agonist, mainly actives DRD2/DRD3/DRD1. Rotigotine extended-release microspheres (RoMS) are a sustained-release formulation that can release sustainably rotigotine for more than 7 days after a single dose of RoMS. This study aimed to investigate whether RoMS can attenuate the lipopolysaccharide (LPS)-induced liver injury of mice. The liver injury was evaluated by assaying serum transaminase and observing histopathological changes. The levels of pro-inflammatory cytokines in serum were also detected. Western blot was employed to assay the expression of proteins in the Akt/NF-κB pathway. The results showed that pre-administration with a single dose of RoMS could inhibit the increase of serum transaminase induced by LPS, alleviate the pathological damage of liver tissue, and decrease the levels of tumor necrosis factor-α and interleukin-6. In addition, RoMS decreased Toll-like receptor 4 protein expression in liver tissue. RoMS mitigated liver injury by activating DRs and negatively regulating the β-arrestin2-dependent Akt/NF-κB signaling pathway. The effects of RoMS could be weakened or abolished by the specific DRD2 antagonist, R121. In conclusion, activation of DRs inhibited the releases of pro-inflammatory cytokines and alleviated the immune-mediated liver injury induced by LPS in mice. The anti-inflammatory mechanism of RoMS may be related to the regulation of the β-arrestin2-dependent Akt/NF-κB signaling pathway.

## Introduction

The liver is an important organ mediating metabolism and immunity. It plays an important role in the phagocytosis of bacteria and pathogens (Jeschke et al., 2007; Jeschke, 2009). Liver injury is a public health problem in the world. Because of its anatomical relationship to the intestinal tract and blood circulation, this allows harmful chemicals, potential pathogens and drug metabolites to enter the liver and induce liver injury (Corazza et al., 2009). Among them, liver injury induced by chemicals, alcohol or drugs is mainly characterized by massive hepatocyte necrosis and structural disorders of liver lobules. The immune system of the liver can produce a rapid immune response to foreign pathogens. When the immune response is excessive, liver immune cells will continue to produce harmful substances such as inflammatory factors, inducing immune-related liver injury.

Lipopolysaccharide (LPS) is a major component of the cell wall of Gram-negative bacteria, inducing inflammatory responses and oxidative stress. LPS-induced liver injury in mice has become a classical model for molecular pharmacology studies and can be used to mimic the process of acute inflammatory response *in vivo* (Tanaka et al., 2015; Koc et al., 2020). Exposure to excess LPS can lead to impaired liver function and hepatocyte degeneration by triggering an inflammatory response. Toll-like receptor 4 (TLR4) is an innate immune sensor for bacterial infection and tissue damage (Chen S. N. et al., 2021). LPS enters the liver through blood circulation and binds to TLR4 on the surface of hepatic macrophages, which in turn can activate the nuclear factor-κB (NF-κB) pathway (Martinon et al., 2009; Wu et al., 2020). This process promotes the production of inflammatory cytokines, chemokines, and inducible nitric oxide synthase, inducing hepatocyte necrosis or apoptosis and accelerating liver injury (Zi et al., 2019). The activated signaling pathways results in an excessive production of inflammatory mediators and the formation of inflammatory factor storms that exacerbates the immune response to LPS-induced liver injury. The difference between LPS-induced immune-mediated liver injury and chemical-induced liver injury is that LPS can disrupt the body’s immune system, inducing an excessive inflammatory response and causing a systemic inflammatory response that can mimic the inflammatory response of sepsis.

Dopamine (DA), a key catecholamine neurotransmitter in the central nervous system, is mainly involved in regulating physiological functions such as movement, mood and cognition (Berke, 2018). Many studies showed a regulatory relationship between DA and the immune system (Pinoli et al., 2017; Xia et al., 2019; Li et al., 2022). It has been shown that almost all immune cells express different dopamine receptors (DRs) on their surface and can synthesize DA on their own to exert immunomodulatory effects in an autocrine or paracrine manner (Xue et al., 2018; Arce-Sillas et al., 2019). DA is involved in regulating innate and adaptive immune responses *in vivo* and *in vitro*, inhibiting the production of pro-inflammatory cytokines, and playing an anti-inflammatory role (Sarkar et al., 2010; Kawano et al., 2018; Vidal and Pacheco, 2020). Meanwhile, some DRs agonists can be used in treating immune-related diseases, such as multiple sclerosis (Melnikov et al., 2021), rheumatoid arthritis (Silvia, 2019), inflammatory bowel disease (Kurnik-Łucka et al., 2021), and Parkinson’s disease, and so on (Zhang et al., 2015). Rotigotine is a non-ergot dopamine receptor agonist that mainly acts on DRD2/DRD3/DRD1. Rotigotine extended-release microspheres (RoMS) is a sustained -release formulation that can release continuously rotigotine for more than 7 days in animals after a single dose of RoMS (Lv et al., 2019; Li et al., 2022). We have previously demonstrated that RoMS alleviated inflammatory pain and alleviated LPS/D-galactosamine-induced acute liver injury in animals (Li T. et al., 2021; Yue et al., 2021). DA and DRs agonists have shown better efficacy in inflammation-related diseases. However, the role and mechanism of continuous DRs activation on LPS-induced liver injury are not clear. Therefore, this study aimed to investigate the effects and potential mechanisms associated with continuous DRs activation on immune-mediated liver injury.

## Materials and Methods

### Animals

Male C57BL/6 mice weighing 22–25 g were purchased from Jinan Pengyue Experimental Animal Breeding Co., Ltd. License number: SCXK (Lu) 20190007. All animal experiments were performed to comply with the National Institutes of Health Guidelines for the Use of Laboratory Animals (Publication 86–23, revised in 1986). The animal study was reviewed and approved by the Ethics Committee of Yantai University (Approval number, YTDX 20180124). All animal studies comply with ARRIVE guidelines.

### Drugs and Chemical Reagents

RoMS (Batch number: 20180902) were provided by Shandong Luye Pharmaceutical Co., Ltd. (Shandong, China). LPS (from *Escherichia coli* 011: B4, Catalog No: L2640) was purchased from Sigma Aldrich (St. Louis, MO, United States). Dexamethasone (DXM) (CAS No.: 50-02-2) was purchased from Sigma Aldrich (St. Louis, MO, United States). R121 (CAS No.:98,185-20-7), a specific DRD2 antagonist, was purchased from Sigma Aldrich (St. Louis, MO, United States). The ELISA kits of TNF-α and IL-6 were provided by Suolaibao, Inc. (Beijing, China). The biochemical kits of AST and ALT were purchased from Nanjing Jiancheng Co., Ltd. (Jiangsu, China). The primary antibodies used in this study are as follows: anti-TLR4 and anti-IL-6 (Santa Cruz Biotechnology, Dallas, TX, United States), anti-dopamine D2 receptor (Abcam, Cambridge, MA, United States), Anti-β-arrestin2, anti-p-Akt, anti-Akt, anti-p-IκBα, anti-IκBα, anti-p-NF-κBp65, anti-NF-κBp65 (Cell Signaling Technology, Danvers, MA, United States), anti-GAPDH (Proteintech Group, Inc., Wuhan, China). BCA protein assay kit and chemiluminescence plus reagents were purchased from Beyotime Biotechnology (Shanghai, China).

### Experimental Protocol

**FIGURE 1:**
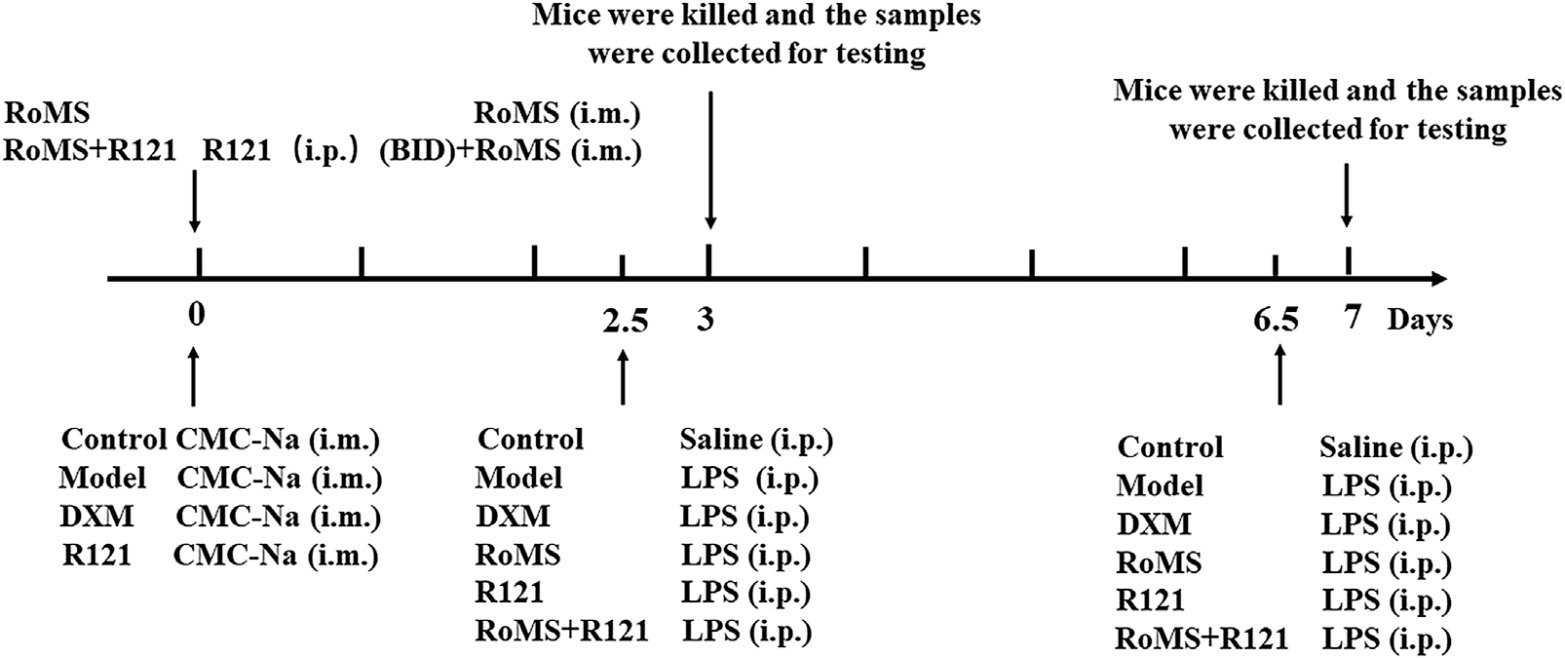
Schematic diagram of the experimental timeline.

A mouse model of liver injury was induced by intraperitoneal injection of LPS (10 mg/kg) as mentioned by the previous study (Chen P. et al., 2021). In this experiment, we chose DXM as a positive reference drug (Jiang et al., 2011; Trivedi et al., 2020). The experimental animals were randomly divided into eight groups (n = 8). The detailed experimental procedures are shown in Figure 1.

1) Control group, intramuscular (i.m.) injection of sodium carboxymethylcellulose (CMC-Na, solvents for RoMS) and intraperitoneal (i.p.) injection of saline; 2) Model group, i. m. injection of CMC-Na and i. p. injection of LPS; 3) DXM group, i. m. injection of CMC-Na and i. p. injection of DXM (5 mg/kg) at 0.5 h before LPS injection; 4) RoMS group, i. m. injection of RoMS 5 mg/kg at 2.5 and 6.5 days before injection of LPS; 5) RoMS group, i. m. injection of RoMS 10 mg/kg at 2.5 and 6.5 days before injection of LPS; 6) RoMS group, i. m. injection of RoMS 20 mg/kg at 2.5 and 6.5 days before injection of LPS; 7) R121 group, i. m. injection of CMC-Na and i. p. injection of R121 (1 mg/kg) at 0.5 h before LPS injection; 8) R121 + RoMS group, i. p. injection of R121 (1 mg/kg) at 0.5 h before RoMS 5 mg/kg injection, twice daily, and i. p. injection of LPS at 2.5 and 6.5 days.

At 12 h after LPS injection, the mice were anesthetized with isoflurane and euthanatized. Blood samples were collected and serum was isolated. The serum was stored at −80°C until analysis. A part of liver tissue was fixed in 4% paraformaldehyde solution. The liver was subsequently stained with hematoxylin and eosin (H&E) staining to evaluate the pathological changes. Another portion was stored at −80°C for western blot assays.

### Histological Examination

The right liver lobe of mice was fixed with 4% paraformaldehyde, embedded in paraffin, and then sectioned. H&E staining was performed. The histological changes of the liver were evaluated by an experimenter who was blinded to the groups under a light microscope.

### Biochemical Assay

The levels of aspartate aminotransferase (AST) and alanine aminotransferase (ALT) in serum were measured by using the commercial kits (C009-2, Nanjing Jiancheng Bioengineering Institute, Nanjing, China).

### Pro-Inflammatory Cytokines Assay

The levels of TNF-α (Lot. 20210415) and IL-6 (Lot. 20210914) in serum were determined by ELISA kits. The sensitivity of the TNF-α kit and IL-6 kit was 18.75 pg/ml. The intra- and inter-assay coefficients of variation were less than 10%.

### Western Blot

The technique of western blotting followed the procedures of the previous study (Yan et al., 2015). The tissue of the liver was placed in a pre-cooled glass grinder. The proteins were extracted with lysis buffer containing PMSF and phosphatase inhibitors. The concentration of protein of each sample was determined by the BCA kit (Beyotime, Shanghai). Proteins were then separated by 10% SDS-PAGE and transferred to a PVDF (0.22 µm) membrane. The membranes were blocked for 4 h in 5% fat-free milk at room temperature. Subsequently, the membranes were washed with TBST buffer three times and incubated overnight at 4°C with specific primary antibodies: anti-TLR4 (1:1,000), anti-DRD2 (1:1,500), anti- β-arrestin2 (1:1,000), anti-p-Akt (1:1,000), anti-Akt (1:1,000), anti-p-IκBα (1:1,000), anti-IκBα (1:1,000), anti-p-NF-κBp65, anti-NF-κBp65 (1:1,000), and anti-GAPDH (1:20,000). Washed with Tris-HCl buffered saline Tween solution, the membranes were probed with HRP-labeled secondary antibody. Bands of proteins were visualized with an ECL plus reagents and gel imager (General Electric Company, United States). The relative protein concentration is analyzed by ImageJ software.

### Statistical Analysis

All data are expressed as the mean ± standard deviation (SD). SPSS 26.0 was used to analyze the data in this study. Tukey’s post-hoc test was performed after one-way analysis of variance. The value of *p* < 0.05 was considered to be significant.

## Results

### Effect of RoMS on the Activities of AST and ALT in Mice

**FIGURE 2:**
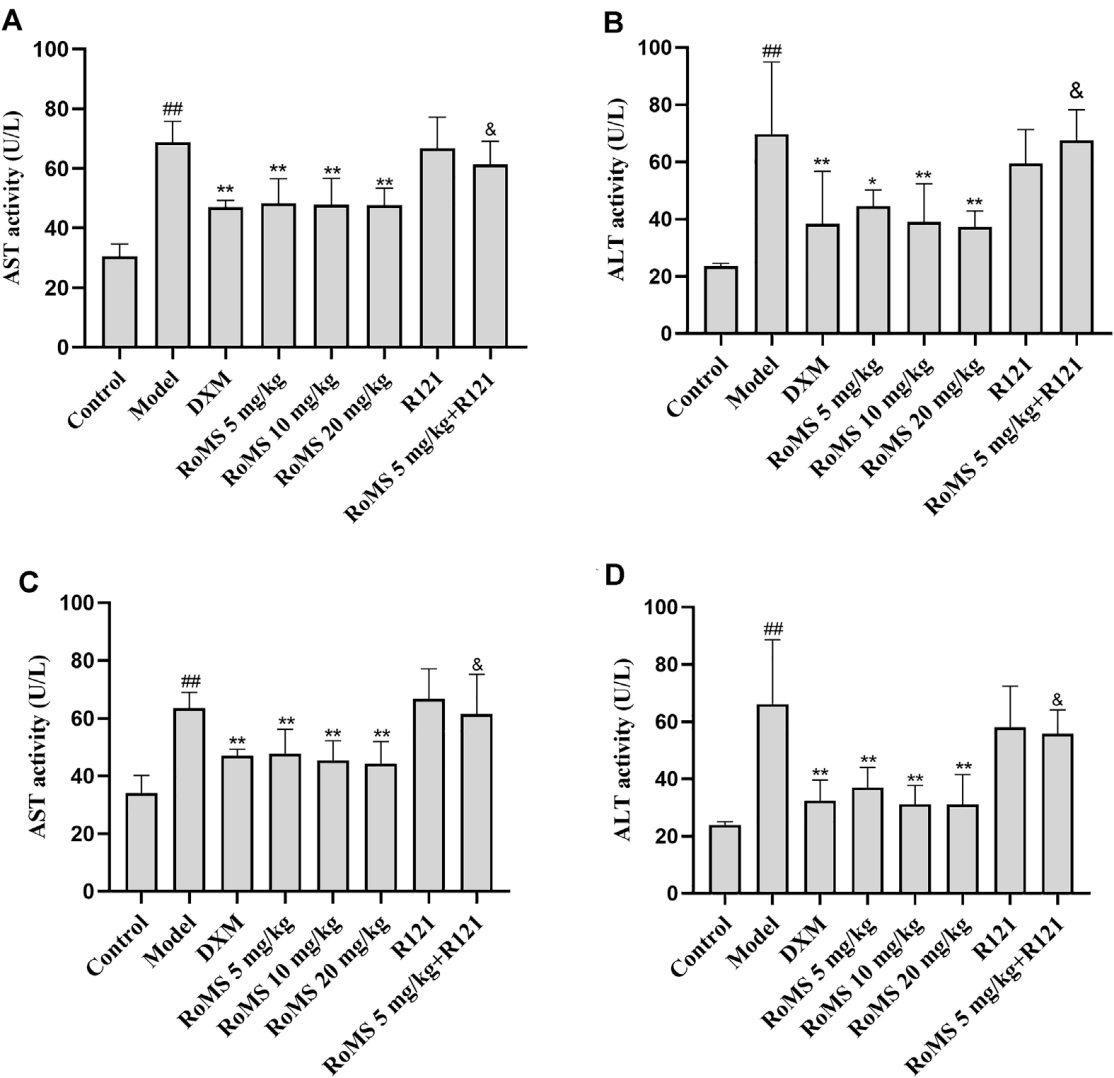
Effects of RoMS on serum aminotransferases activities. At 3 days after RoMS pre-administration **(A,B)**. At 7 days after RoMS pre-administration **(C,D)**. The data are expressed as the mean ± SD (n = 8 in each group). ^##^ *p* < 0.01 versus the control group, *p < 0.05 or **p < 0.01 versus the model group, ^&^ *p* < 0.05 or ^&&^ *p* < 0.01 versus RoMS 5 mg/kg group.

To evaluate the effect of RoMS on immune-mediated liver injury, the activities of AST and ALT in serum were measured at 3 and 7 days after RoMS pre-administration. In the model group, the activities of AST (Figures 2A,C) and ALT (Figures 2B,D) levels were elevated after LPS injection. Compared with the model group, the activities of AST and ALT were decreased after DXM treatment. The activities of AST and ALT were also reduced at 3 and 7 days after RoMS pre-administration in a dose-dependent manner. R121, a DRD2-specific antagonist, had no significant effect on the activities of AST and ALT. R121 abrogated partially the effects of RoMS on the activities of AST and ALT. These results suggest that RoMS can attenuate immune-mediated liver injury.

### Effect of RoMS on Liver Histopathological Changes in Mice

**FIGURE 3:**
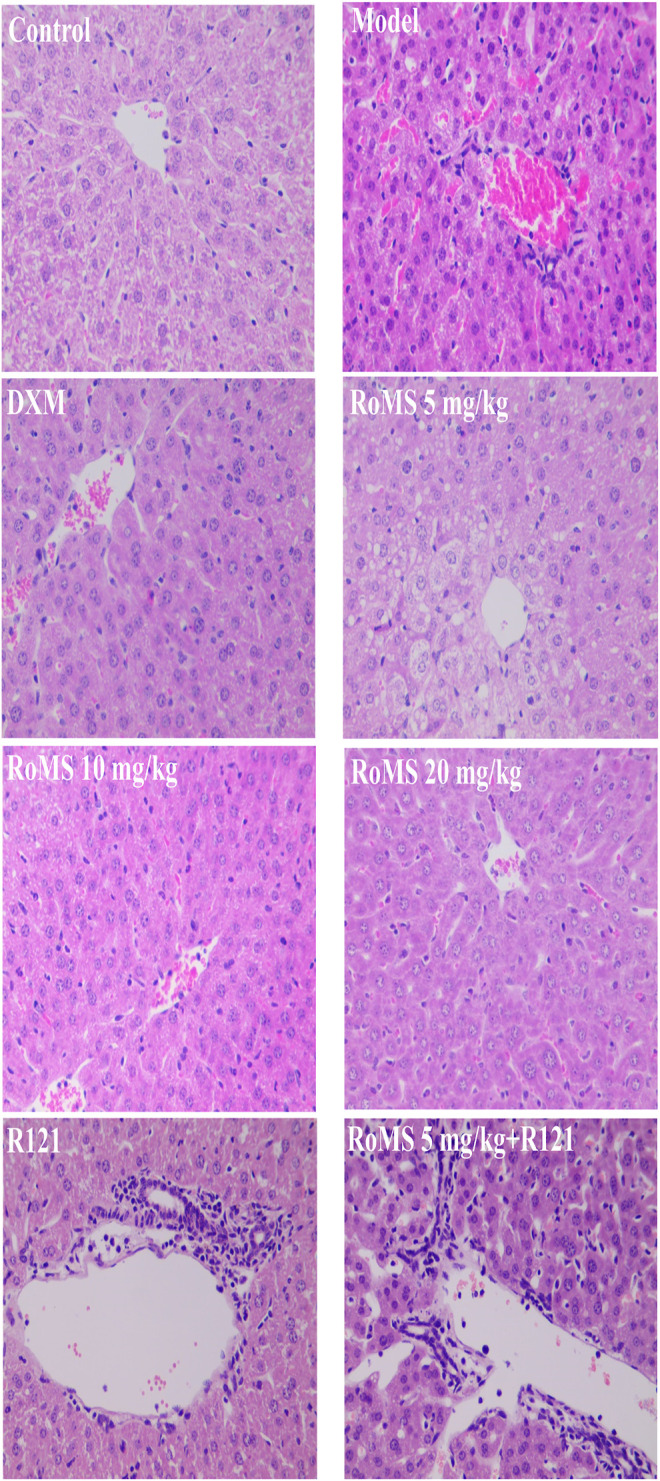
Effect of RoMS (at 3 days after pre-administration) on liver injury in mice. Liver tissues were stained with H&E, ×400 magnification.

**FIGURE 4:**
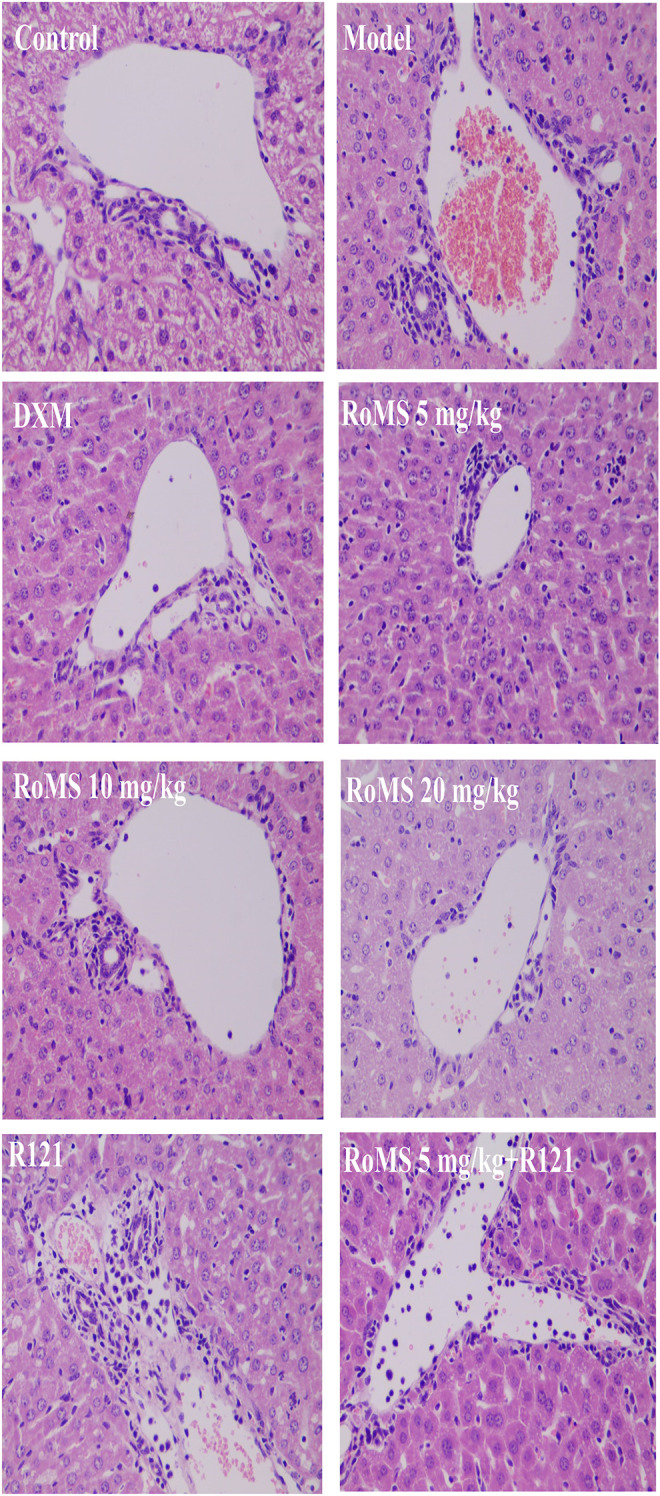
Effect of RoMS (at 7 days after pre-administration) on liver injury in mice. Liver tissues were stained with H&E, ×400 magnification.

Histopathological examination showed no abnormalities in the liver tissue of control mice. The liver of the mice in the model group showed abnormal structure, such as extensive hemorrhage, massive hepatocellular edema, and inflammatory cell infiltration. Compared with the model group, these abnormalities of the liver were significantly improved at 3 (Figure 3) and 7 days (Figure 4) after RoMS pre-administration, such as a slight hemorrhage, a decreased inflammatory cell infiltration, and partial hepatocellular edema in the portal area and around the central vein. No improvement of liver injury in the mice of the R121 group was observed. Compared with the RoMS 5 mg/kg group, the liver tissue damage in the mice of RoMS plus R121 group was aggravated.

### Effect of RoMS on the Levels of TNF-α and IL-6 in Mice

**FIGURE 5:**
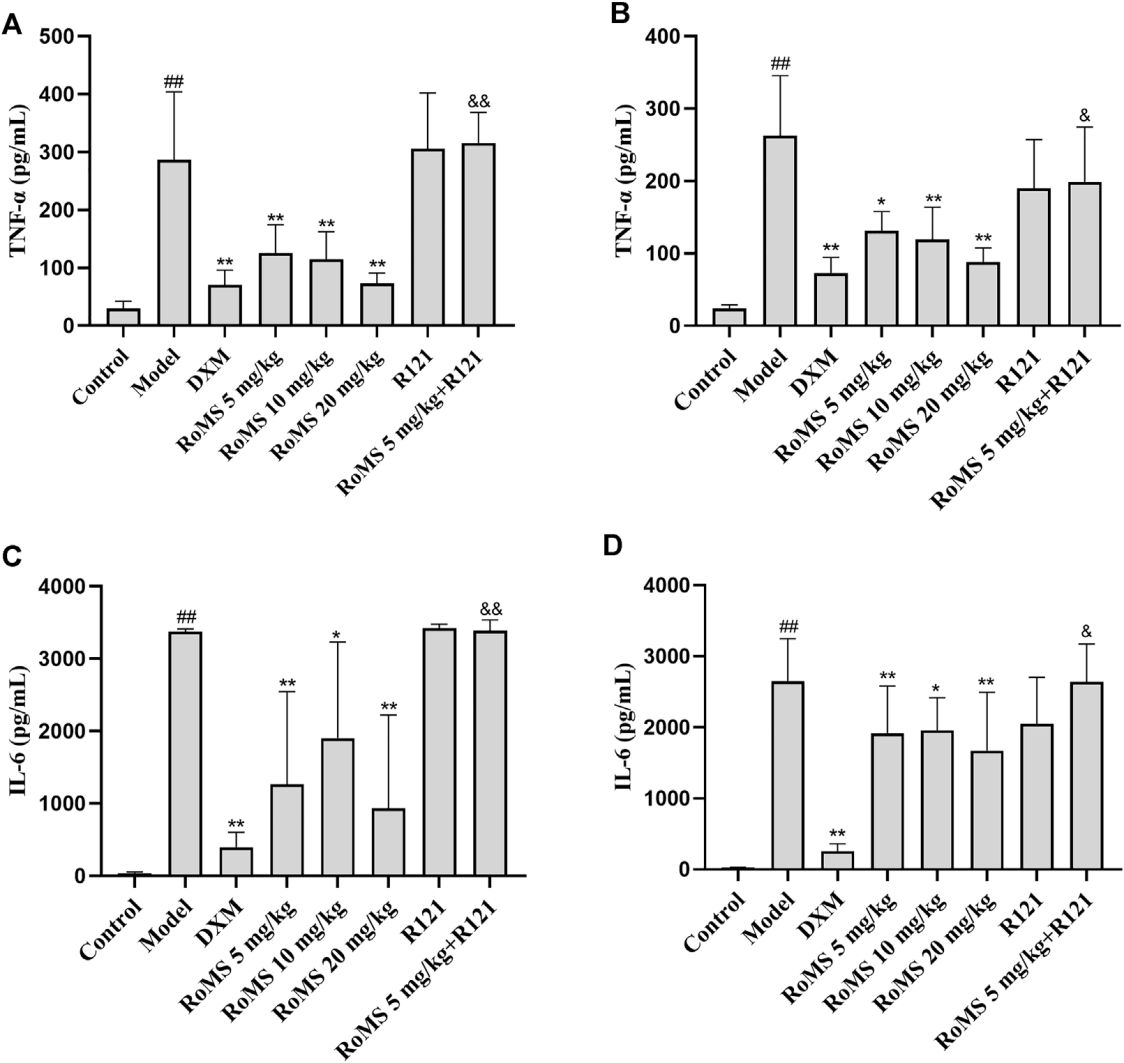
Effect of RoMS on the levels of TNF-α and IL-6 in septic mice. At 3 days after RoMS pre-administration **(A,C)**. At 7 days after RoMS pre-administration **(B,D)**. The Data are expressed as the mean ± SD (n = 8 in each group). ^##^ *p* < 0.01 versus the control group, *p < 0.05 or **p < 0.01 versus the model group, ^&^ *p* < 0.05 or ^&&^ *p* < 0.01 versus RoMS 5 mg/kg group.

To investigate the potential anti-inflammatory effects of RoMS, the levels of TNF-α and IL-6 in serum were monitored. Compared with the control group, the levels of TNF-α and IL-6 in the model group increased sharply after LPS exposure. Compared with the model group, the levels of TNF-α and IL-6 were significantly decreased in the DXM group. At 3 (Figures 5A,C) and 7 days (Figures 5B,D) after RoMS pre-administration, the levels of TNF-α and IL-6 were also reduced. The RoMS-mediated decrease of the levels of TNF-α and IL-6 were abrogated by R121 administration. These results suggest that RoMS can continuously activate DRs, inhibiting the secretion of the pro-inflammatory cytokines, and therefore attenuating the LPS-induced inflammatory response.

### Effect of RoMS on the Akt/NF-κB Pathway in the Liver of Mice

**FIGURE 6:**
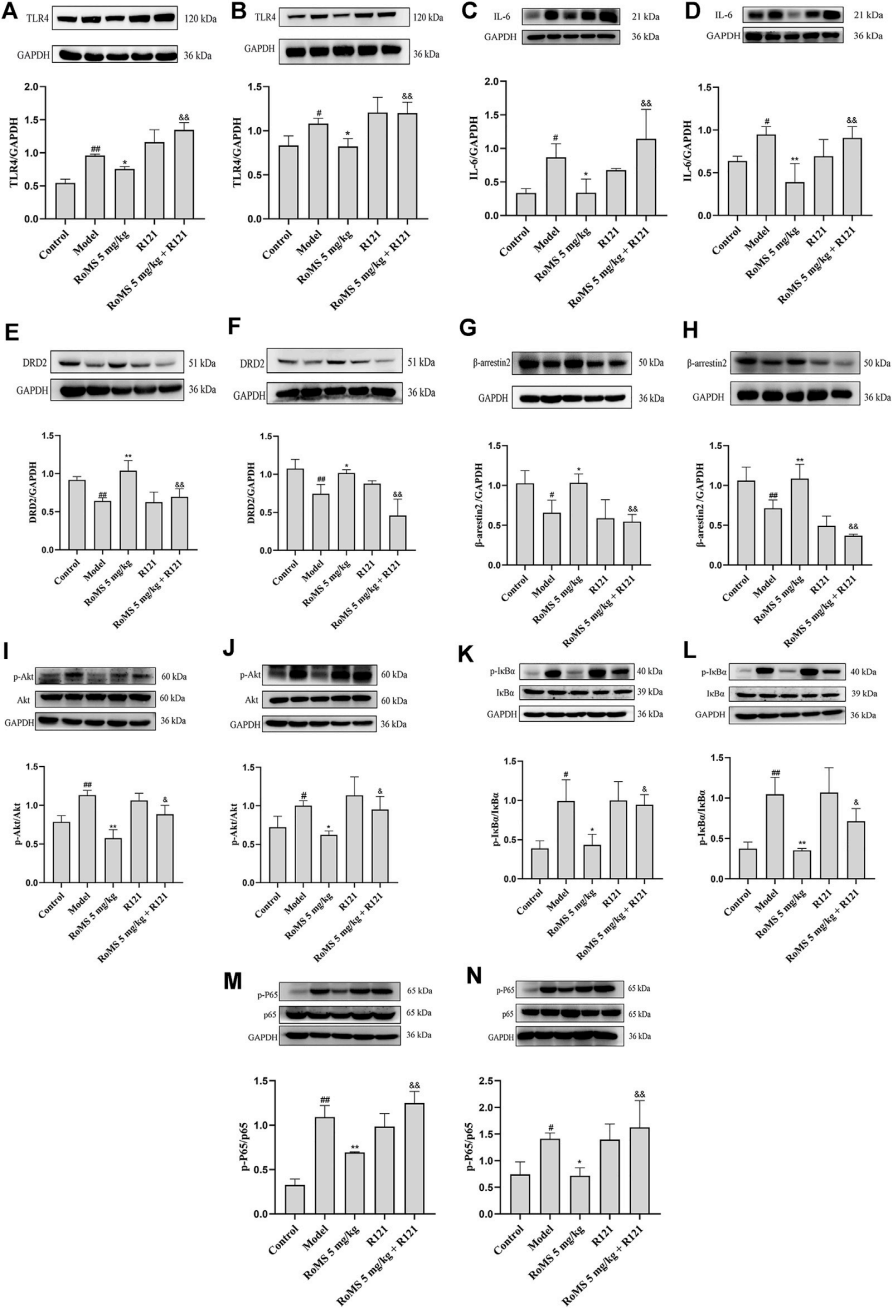
Effects of RoMS on the Akt/NF-κB pathway related proteins in the liver of mice. At 3 d and 7 days after RoMS pre-administration, the levels of TLR4 **(A,B)**, IL-6 **(C,D)**, DRD2 **(E,F)**, β-arrestin 2 **(G,H)**, p-Akt/Akt **(I,J)**, p-IκBα/IκBα **(K,L)**, p-P65/p65 **(M,N)**. The data are expressed as the mean ± SD (n = 3 in each group). ^#^ *p* < 0.05 or ^##^ *p* < 0.01 versus control group, *p < 0.05 or **p < 0.01 versus model group, ^&^ *p* < 0.05 or ^&&^ *p* < 0.01 versus RoMS 5 mg/kg group.

Next, we investigated the expression of proteins of the Akt/NF-κB pathway in the liver tissue of mice. The results showed that the expression of TLR4 (Figures 6A,B) and IL-6 (Figures 6C,D) were elevated after LPS injection, which is consistent with previous findings (Chen S. N. et al., 2021). Pre-administration with RoMS reduced the expression of TLR4 and IL-6 in liver tissue. While R121 administration blocked the effect of RoMS on the expression of TLR4 and IL-6. Compared with the control group, the levels of phosphorylation of Akt (Figures 6I,J), IκBα (Figures 6K,L), and p-P65 (Figures 6M,N) were increased in the liver tissues after LPS exposure. However, the levels of phosphorylation of Akt, IκBα, and p-P65 were decreased by RoMS pre-administration. Consistent with the previous results, R121 abolished partially the RoMS-mediated effects on the expression of the proteins of the Akt/NF-κB pathway. In line with previous studies (Du et al., 2018), the LPS-induced liver injury reduced the expression of DRD2 (Figures 6E,F) and β-arrestin2 (Figures 6G,H). However, RoMS increased the expression of DRD2 and β-arrestin2. R121 abolished the effect of RoMS on the expression of DRD2 and β-arrestin2 in the mice. These results suggested that β-arrestin2 combining G protein-coupled receptors plays a role in the RoMS-regulated Akt/NF-κB pathways.

## Discussion

LPS-induced liver injury is involved in the development of several liver diseases. And Kupffer cells play an important role in the processes of LPS-induced liver injury. LPS is a typical immune activator. It is also a well-studied pathogen-associated agent that induces systemic inflammatory response syndrome. LPS can lead to multiple organ dysfunction and is life-threatening if left unchecked. This study is carried out by a mouse model of LPS-induced inflammatory liver injury.

LPS binds to TLR4 on the surface of Kupffer cells, further activating the Akt/NF-κB signaling pathway. NF-κB, a nuclear transcription factor with a broad regulatory role, plays an important role in regulating the intrinsic host immune response. When the stimulatory signal from LPS is delivered to the IKK kinase, the NF-κB inhibitory protein IκB-α in the cell plasma is phosphorylated and ubiquitinated and then degraded via the proteasome pathway. NF-κB rapidly enters the nucleus to initiate and regulate the transcription of genes related to the immune response, inflammatory response (Feng et al., 2022). Pro-inflammatory cytokines play a key role in the acute phase of inflammation response and are key mediators in the process of liver inflammatory injury (Li Q. et al., 2021). LPS can cause a series of liver pathological changes, such as severe hemorrhagic necrosis, massive inflammatory cell infiltration, and structural destruction of liver lobules. AST and ALT are the most sensitive indicators of liver injury. In the present study, we found that the levels of AST and ALT were elevated in the serum of mice at 12 h after LPS injection. H&E staining showed that the liver tissue of mice in the model group showed large hepatocellular edema, necrosis and inflammatory cell infiltration.

In addition, the levels of pro-inflammatory cytokines TNF-α and IL-6 were significantly increased in the serum of mice after LPS injection. This suggests that LPS can cause inflammatory liver injury in mice. Our results indicated that pro-inflammatory cytokines levels could be significantly suppressed during 3 and 7 days of pre-administration of RoMS. However, this protective effect of RoMS could be partially abolished when the DRD2-specific blocker R121 was administered. Taken together, these results show that RoMS could attenuate the LPS-induced inflammatory response in mice by activating DRs.

The previous study in our lab showed that rotigotine and RoMS had a protective effect on the LPS/D-galactosamine-induced acute liver failure, which is consistent with the main manifestations of clinical acute liver failure (Nakama et al., 2001; Yue et al., 2021). There are differences between LPS-induced liver injury and LPS/D-galactosamine-induced liver injury. D-galactosamine is a hepatocyte-specific chemical toxicant. D-galactosamine can irreversibly bind to uracil triphosphate nucleoside (UTP) and cause UTP depletion. As a result, it inhibits of UTP-dependent synthesis of nucleic acids, glycoproteins and other substances and then leads to organelle and cell membrane damage, and extensive apoptosis or necrosis of hepatocytes. Therefore, the LPS/D-galactosamine-induced acute liver failure model is commonly used for drug screening (Xia et al., 2014).

There is increasing evidence that DA acts as an immunomodulatory molecule when DA binds to DRs on peripheral immune cells (Matt and Gaskill, 2020). Previous studies have demonstrated that DA can activate DRD1 of macrophages and exert anti-inflammatory effects by inhibiting NLRP3 (Liu and Ding, 2019). RoMS is a long-acting extended-release preparation of rotigotine that allows for a sustained release of rotigotine for more than 7 days (Wang et al., 2012). RoMS mainly activates DRD1/DRD2/DRD3 (Wood et al., 2015). Continuous DRs activation was achieved by pre-administration of RoMS at 3 and 7 days. Hence, we investigated the effect of continuous DRs activation on acute inflammatory responses *in vivo*. The results revealed that RoMS could reduce the transaminase levels, inhibit the production of TNF-α and IL-6, and alleviate hepatic histopathological damage in mice at 3 or 7 days after a single administration of RoMS. The results mentioned above are consistent with our previous study showed that the blood concentration of rotigotine was below the detection limit at 14 days after a single treatment with RoMS (Lv et al., 2019). These results suggest that the continuous DRs activation produced by RoMS pre-administration can exert a sustained anti-inflammatory effect.

**FIGURE 7:**
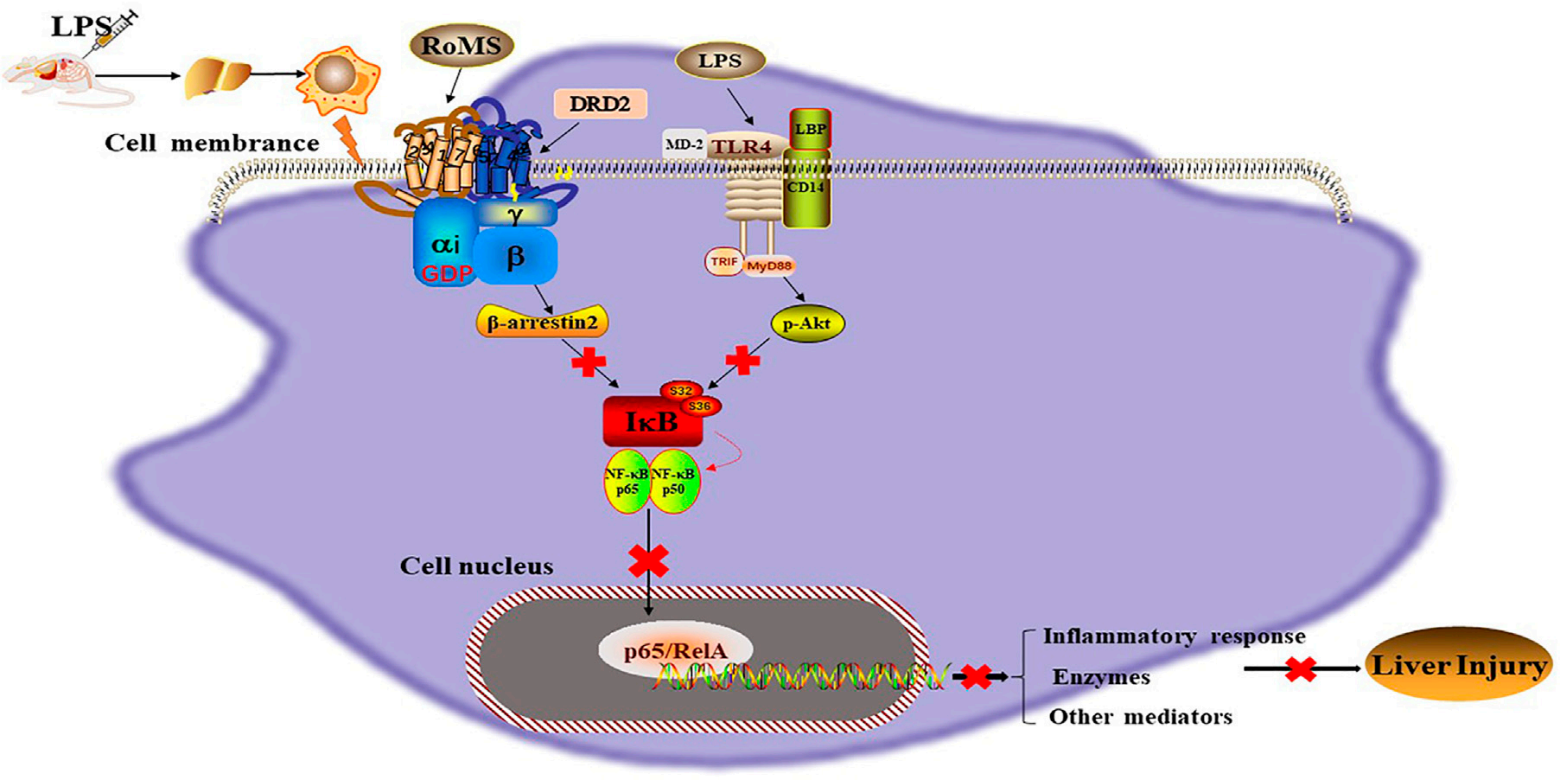
Mechanism of RoMS in immune-mediated liver injury. LPS binds to TLR4 on the surface of hepatic Kupffer cells and promotes the phosphorylation levels of Akt, IκBα and NF-κBp65, which drives NF-κBp65 into the nucleus to regulate the transcription of target genes. This can induce excessive inflammatory responses and secretion of multiple pro-inflammatory cytokines. The results show that RoMS can activate DRs on the surface of hepatic Kupffer cells and negatively regulate the Akt/NF-κB pathway through a β-arrestin2-dependent mechanism, thereby attenuating LPS-induced inflammatory liver injury. RoMS: Rotigotine extended-release microspheres; LPS: Lipopolysaccharide; TLR4: Toll-like receptor 4; MD2: myeloid differentiation 2; TRIF: TIR domain-containing adapter inducing interferon-β; LBP: LPS binding protein; IκB: Inhibitor of kappa B; NF-κBp65: Nuclear factor-kappa B; GDP: Guanosine diphosphate; β-arrestin2: β-inhibitor protein 2; Akt: Protein kinase B; MyD88: Myeloiddifferentiationfactor88.

A previous study found that the DRD5-ARRB2-PP2A signaling pathway could block the TRAF6-mediated NF-κB pathway, thereby attenuating the systemic inflammatory response and reducing the mortality of septic mice (Wu et al., 2020). In addition, Han and colleagues demonstrated that DRD2 activation inhibits the inflammatory response in acute pancreatitis through the PP2A-dependent Akt/NF-κB pathway (Han et al., 2017; Ye et al., 2020). These results suggest that the NF-κB signaling pathway plays a vital role in the inflammatory response. The translocation of p-P65 into the nucleus can promote the release of pro-inflammatory cytokines as well as other mediators, which then induce a strong inflammatory response (Park et al., 2015). The acute inflammatory response promotes the recruitment and over-activation of immune cells, synthesizing and secreting more inflammatory factors, inducing a cascade amplification of inflammation, and triggering an “inflammatory storm”. The inflammatory storm leads to a dysregulation of the body’s immune system and even to a septic shock and a multi-organ failure. LPS binding to TLR4 promotes the increase of phosphorylation of Akt, I-κBα, and p65, which promotes the release of high levels of inflammatory cytokines. In the present study, a mouse model of immune-mediated liver injury was induced by intraperitoneal injection of LPS. Pre-administration of RoMS inhibited the expression of TLR4, suppressed the phosphorylation levels of Akt, I-κBα, and p-P65, and therefore decreased the levels of inflammatory factors. Furthermore, we found that the expression of DRD2 and β-arrestin2 of hepatic immune cells was reduced after LPS injection. Activation of DRs alleviated the immune-mediated liver injury by regulating the Akt/NF-κB pathway through binding to β-arrestin2. In line with our hypothesis, the anti-inflammatory effects of RoMS were blocked partially by the DRD2-specific blocker R121. Taken together, the results of this study provide preliminary evidence for the anti-inflammatory effect of DRs in immune-mediated liver injury. Overall, the continuous activation of DRs promoted the expression of β-arrestin2, which inhibited the NF-κB signaling pathway and decreased the production of inflammatory mediators (Figure 7). Compared to previous studies, the novelty of this study is that the continuous activation of DRs achieved by RoMS can effectively attenuate the immune-mediated liver injury, which is different from the acute liver failure induced by LPS/D-galactosamine.

There is a limitation in the present study. Due to the properties of RoMS on the DRs, we cannot completely exclude the effects of RoMS on the other subtypes of DRs.

## Conclusion

The present study showed that RoMS can provide a protective effect in the LPS-induced liver injury via the activation of DRs. Activation of DRs alleviates the inflammatory liver injury by regulating the Akt/NF-κB pathway through binding to β-arrestin2. This finding suggests that DRs may be a target to exert anti-inflammatory effects. However, further *in vitro* and *in vivo* studies are needed before DRs agonists are used clinically for the treatment of inflammatory diseases.

## Data Availability

The original contributions presented in the study are included in the article/Supplementary Material, further inquiries can be directed to the corresponding authors.
